# The New Radiolabeled Peptide ^99m^TcEDDA/HYNIC-TOC: Is It a Feasible Choice for Diagnosing Gastroenteropancreatic NETs?

**DOI:** 10.3390/cancers14112725

**Published:** 2022-05-31

**Authors:** Mirela Gherghe, Alexandra Maria Lazăr, Adina Elena Stanciu, Mario-Demian Mutuleanu, Maria-Carla Sterea, Cristina Petroiu, Laurenția Nicoleta Galeș

**Affiliations:** 1 Nuclear Medicine Department, University of Medicine and Pharmacy “Carol Davila” Bucharest, 050474 Bucharest, Romania; mirela.gherghe@umfcd.ro (M.G.); mario-demian.mutuleanu@drd.umfcd.ro (M.-D.M.); 2 Nuclear Medicine Department, Institute of Oncology “Prof. Dr. Alexandru Trestioreanu”, 022328 Bucharest, Romania; maria-carla.sterea@rez.umfcd.ro (M.-C.S.); cristinacos@yahoo.com (C.P.); 3 Carcinogenesis and Molecular Biology Department, Institute of Oncology “Prof. Dr. Alexandru Trestioreanu”, 022328 Bucharest, Romania; adinaelenastanciu@yahoo.com; 4 Oncology Department, University of Medicine and Pharmacy “Carol Davila” Bucharest, 050474 Bucharest, Romania; laurentia.gales@umfcd.ro; 5 Oncology Department, Institute of Oncology “Prof. Dr. Alexandru Trestioreanu”, 022328 Bucharest, Romania

**Keywords:** ^99m^TcEDDA/HYNIC-TOC, gastroenteropancreatic neuroendocrine tumours, ^68^Ga-DOTA-peptides, somatostatin analogs

## Abstract

**Simple Summary:**

Gastroenteropancreatic neuroendocrine tumors are a group of heterogenous tumors that account for about 60% of the neuroendocrine tumors diagnosed nowadays. They possess the capability to synthesize and secrete peptides and hormones that lead to their characteristic syndromes. One of their main characteristics is the expression of somatostatin receptors, which makes them appropriate for diagnosis based on radiolabeled somatostatin analogs scintigraphy and PET/CT. This retrospective monocentric study was conducted to determine the suitability of ^99m^TcEDDA/HYNIC-TOC scintigraphy in diagnosis and management of gastroenteropancreatic neuroendocrine tumors, in comparison with other available radiotracers. We examined 173 patients referred to our clinic for a ^99m^TcEDDA/HYNIC-TOC scan and, based on the obtained results, we estimated the sensitivity, specificity, accuracy, and positive and negative predictive values of the method. Our results confirm the utility of ^99m^TcEDDA/HYNIC-TOC in the management and follow-up of patients suffering from gastroenteropancreatic neuroendocrine tumors, especially in centers that do not benefit from PET/CT equipment or ^68^Ga-DOTA-peptides.

**Abstract:**

(1) Background: The aim of our study is to reveal the advantages and limitations of the use of ^99m^TcEDDA/HYNIC-TOC (Tektrotyd^®^, Polatom) in the diagnosis of gastroenteropancreatic neuroendocrine tumors and to compare our results with the values obtained for ^111^In-pentetreotide and ^68^Ga-DOTA-peptides, routinely used in medical practice. (2) Methods: This retrospective monocentric study included 173 patients with gastroenteropancreatic neuroendocrine tumors who underwent ^99m^TcEDDA/HYNIC-TOC scans as part of their clinical management. The examination protocol included a whole-body scan acquired 2 h after the radiotracer’s administration, with the SPECT/CT performed 4 h post-injection. Physiological and abnormal uptake were established by two experienced physicians and, based on the obtained results, sensitivity, specificity, accuracy, positive predictive value and negative predictive value were calculated. (3) Results: Our method presented a sensitivity of 90.5%, a specificity of 71.9%, and an accuracy of 84.3%, with a positive predictive value of 86.7% and a negative predictive value of 78.8%. (4) Conclusions: ^99m^Tc-EDDA/HYNIC-TOC, a receptor-based radiopharmaceutical, could represent a competitor for ^68^Ga-labeled peptides in the diagnosis and management of patients with gastroenteropancreatic neuroendocrine tumors. Our results show a lower sensitivity (90.5%) than ^68^Ga-DOTA-peptides, but with great specificity, accuracy, positive, and negative predictive values.

## 1. Introduction

Neuroendocrine tumors (NETs) are a group of heterogeneous tumors that originate from diffused neuroendocrine cells dispersed throughout the body, more specifically in the gastrointestinal tract, the islets of Langerhans in the pancreas, and the bronchopulmonary system. They possess the unique capability to synthesize and secrete peptides and hormones that lead to their characteristic syndromes; however, it is not uncommon to be asymptomatic and discovered incidentally [1].

Gastroenteropancreatic NETs (GEP-NETs) account for about 60% of the NETs [2]. The latest update on the World Health Organization (WHO) classification on the tumors of the digestive system, made in 2019, introduces GEP-NETs as neuroendocrine neoplasms (NENs), describing two major categories: one of the well-differentiated NETs that were previously described as carcinoid tumors of the digestive tract, and a second category of neuroendocrine carcinomas (NECs) that are poorly-differentiated and, although positive for neuroendocrine markers, have a poor evolution and prognosis [3,4,5].

GEP-NETs may present as hormonally functional or nonfunctional tumors and may have distinct clinical features based on their site of origin. Additionally, they present a specific tissue characteristic that can be targeted by molecular imaging and peptide receptor radionuclide therapy (PRRT), such as expression of different receptors, especially somatostatin receptors (SSTRs), particularly SSTR2 and SSTR5 [6,7,8,9,10].

The diagnostic algorithm for GEP-NETs is based on five main pillars: morphological imaging, endoscopic procedures, pathology, functional imaging, and circulating biomarkers [11]. Functional imaging studies are based on the ability of NETs to overexpress SSTRs on their cell surface [12]. Somatostatin receptor imaging includes SPECT/CT and PET/CT acquisitions with adequate radiotracers that allow for accurate delineation of disease extent at both initial staging and follow-up and help identify even an occult primary tumor [2]. Over the years, many radiolabeled somatostatin analogs have been developed for human use, with variable sensitivity and specificity, since they differ in terms of radionuclides, chelators, and affinity for different SSTR subtypes [13,14]. For many years, somatostatin receptor scintigraphy (SRS) with ^111^In-DTPA-DPhe^1^-octreotide [^111^In-pentetreotide] (Octreoscan^®^) has been considered the “golden standard” for diagnosis and monitoring of GEP-NETs. However, research on new radiopharmaceuticals labeled with ^99m^Tc and ^68^Ga, which have a higher uptake in the target tissue, added to the improvement in resolution of the new SPECT and PET scanners, have limited the use of ^111^In-pentetreotide SRS and favored the use of these radiotracers that had better imaging characteristics [15,16,17]. ^99m^Tc-labelled peptides have been recently introduced in the practical clinic and demonstrated some advantages compared with ^111^In-pentetreotide, having shown higher sensitivity, better imaging quality, and lower radiation exposure for patients, which can make them an alternative to ^68^Ga-DOTATOC examinations [13]. The use of ^99m^TcEDDA/HYNIC-TOC could represent a good alternative to ^68^Ga-labeled peptides in hospitals and centers that do not own PET scanners or ^68^Ge/^68^Ga generators [18]. ^99m^TcEDDA/HYNIC-TOC has a higher affinity for SSTR2 and lower affinity for SSTR3 and SSTR5 [19]. The use of the SPECT/CT system significantly improves the specificity, sensitivity, and accuracy of SSTR SPECT-alone studies and could represent an opportunity for quantitative assessment of the radiotracer’s uptake in different organs and lesions [18,20].

Our study aims to reveal the advantages and limitations of the use of ^99m^TcEDDA/HYNIC-TOC (Tektrotyd^®^, Polatom, Otwock, Poland) in the diagnosis of GEP-NETs and to compare our results with the ones obtained for other radiolabeled peptides routinely used for SPECT/CT and PET/CT imaging.

## 2. Materials and Methods

### 2.1. Patient Selection

Our study included retrospective data from consecutive patients who were referred to the Nuclear Department of Oncology Institute “Prof. Dr. Alexandru Trestioreanu”, Bucharest, Romania, for a ^99m^TcEDDA/HYNIC-TOC scan as part of their clinical management, between November 2015 and March 2022. The analysis was approved by the local ethics committee of our institution. Due to the retrospective nature of our study, supplementary written consent from the patients was not necessary. Out of the 251 investigations performed during this period, we selected 173 patients with either histopathologically confirmed GEP-NETs (146 patients) or biochemical markers and/or imaging findings raising the suspicion of NETs (27 patients). Immunohistochemical tumoral markers and serum biochemical markers, such as somatostatin receptors (SSTR), Chromogranin A (CGA), Synaptophysin (SYN), CD56, CDX2, proliferation index KI-67, Neuron Specific Enolase (NSE), Serotonin, Insulin, Glucagon and Gastrin, as well as urinary 5-hydroxyindoleacetic acid (5-HIAA), were collected from patients’ medical records. Patients under treatment with long-acting release somatostatin analogs were requested to stop their administration at least 4 weeks prior to the investigation.

### 2.2. ^99m^Tc-EDDA/HYNIC-TOC Synthesis

All reagents and solvents were purchased from commercial suppliers and used with no other purification. Technetium (^99m^Tc) was obtained from a ^99^Mo/^99m^Tc radionuclide generator system (Ultra-Technekow FM Generator produced by Curium Netherlands BV). ^99m^Tc decays by gamma emission (energy 141 keV), with a physical half-life of 6.02 h to technetium-99.

The preparation of ^99m^Tc-EDDA/HYNIC-TOC, consisting of radiolabeling of HYNIC-[D-Phe1Tyr3-Octreotide] trifluoroacetate with ^99m^Tc, was carried out manually in a preheated reaction vial at 80 °C, according to the manufacturer’s instructions, just before the intravenous administration. The peptide HYNIC-[D-Phe1Tyr3-Octreotide] trifluoroacetate was provided by IAE POLATOM (Otwock, Poland) in the commercial Tektrotyd^®^ kit.

### 2.3. ^99m^Tc-EDDA/HYNIC-TOC Radiochemical Purity

The radiochemical purity of the ^99m^Tc-EDDA/HYNIC-TOC was checked using radio thin-layer chromatography (radio-TLC) before each patient’s administration. In applications where there are few radioactive species, radio-TLC is preferred over radio-high-performance liquid chromatography due to its simplicity and relatively quick analysis time [21]. The radiochemical purity of the administered radiopharmaceutical ^99m^Tc-EDDA/HYNIC-TOC was higher than 95%.

### 2.4. Acquisition Technique and Reconstruction

Subjects were recommended increased hydration, light food intake, and mild laxatives at least 24 h prior to the investigation for a better visualization of the abdominal region. They were asked to not consume any food, simple carbohydrates or liquids other than plain water for at least 6 h prior to the examination.

Patients were administered a dose of 628–740 MBq (17–20 mCi) of ^99m^Tc-EDDA/HYNIC-TOC intravenously and “whole-body” (WB) planar scans and SPECT/CT acquisitions were performed. The examination protocol included a WB scan acquired 2 h after the radiotracer’s administration, with the SPECT/CT performed 4 h post-injection. The examinations were performed using a dual-head Discovery 670 DR SPECT/CT system (General Electric Healthcare, Chicago, IL, USA) with low-energy, high-resolution (LEHR) collimators. The whole-body planar scan was acquired using the following parameters: pallet velocity of 10 cm/min, 128 × 128 matrix size, zoom of 1.0× and energy window of 140.5 keV ± 10%. SPECT/CT imaging of the chest, abdomen, and pelvis was performed using a standard protocol: 128 × 128 matrix size, step and shoot rotation time of 30 s/view, for a total of 120 views, dual energy window (140 keV ± 10% and 120 keV ± 5%). After the SPECT acquisition, a low-dose CT scan was performed, maintaining the position of the patient at 120 keV, 40–340 mA, dose modulation enabled (GE Smart Scan). CT images were acquired with a 3.75 slice thickness and then reconstructed using the standard, bone plus, and lung reconstruction filters, with slice thicknesses of 2.5 mm, 1.25 mm, and, respectively, 1.25 mm. Patients with a suspicion of gastric NETs were asked to drink water right before the SPECT/CT examination, to distend the stomach for a better visualization on the CT scan.

SPECT/CT data were reconstructed using the Q.Volumetrix software (GE Xeleris 4.0, General Electric Healthcare, Chicago, IL, USA) provided by the manufacturer, using the ordered subset expectation maximization (OSEM) iterative reconstruction algorithm, with 8 subsets and 10 iterations, resolution recovery, attenuation correction, scatter correction, Butterworth filtering.

### 2.5. Image Analysis

**Figure 1:**
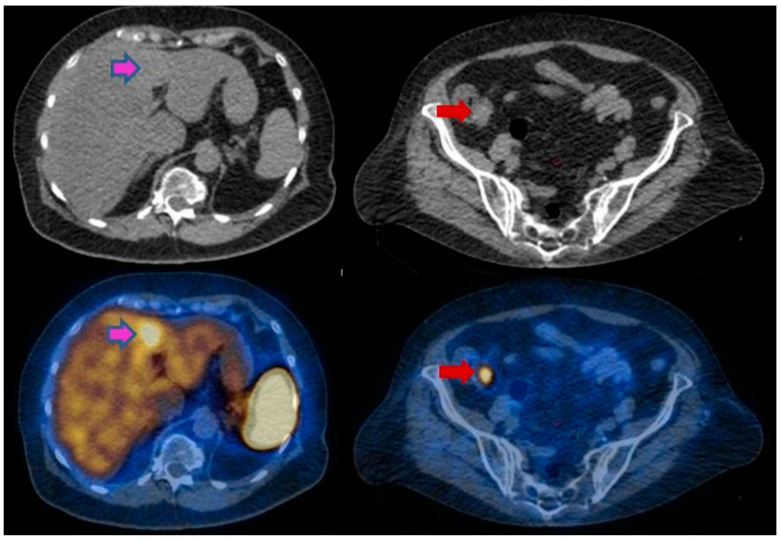
A 71-year-old female patient, diagnosed with a hepatic metastasis of neuroendocrine tumor of unknown origin (confirmed by biopsy, NET G1). The SPECT/CT images show the presence of the hepatic lesion (pink arrows), also showcasing the primary tumor in the ileum (red arrow). The histopathological exam performed after surgical excision of the mass confirmed the presence of a neuroendocrine tumor.

The obtained images were interpreted independently by two experienced physicians on a dedicated workstation for imaging diagnosis (GE Xeleris 4.0, General Electric Healthcare, Chicago, IL, USA). Normal physiological ^99m^Tc-EDDA/HYNIC-TOC uptake was established in organs using radiological images (CT) and clinical correlations (thyroid, spleen, liver, kidneys and bladder). Abnormal uptake was determined in primary lesions and metastatic disease sites, such as lymph nodes, liver, bone, lung, and adrenal glands. Areas that demonstrated increased ^99m^Tc-EDDA/HYNIC-TOC activity were correlated to their morphological correspondent on the CT images (Figure 1).

### 2.6. Statistical Analysis

Data analysis was performed using IBM SPSS Statistics Version 26 (IBM, SPSS, Inc., Chicago, IL, USA, 2019). The results were presented as frequency (percent) and mean ± standard deviation. Diagnostic performance of ^99m^Tc-EDDA/HYNIC-TOC was determined using the Crosstabs function, resulting in the sensitivity, specificity, accuracy, positive predictive value (PPV), and negative predictive value (NPV).

## 3. Results

**Figure 2:**
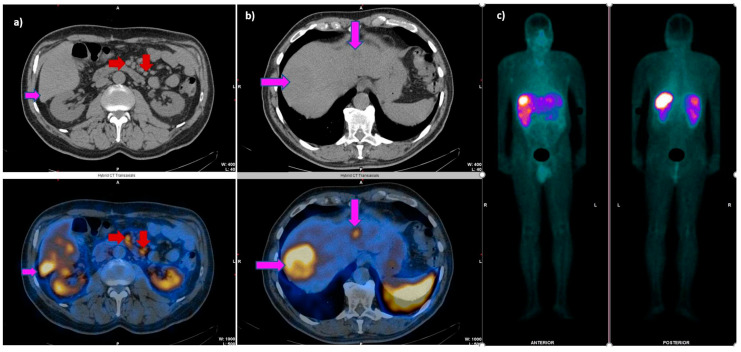
A 64-year-old male patient diagnosed with an ileal neuroendocrine tumor (NET G1), excized in 2021. The present SRS shows increased uptake corresponding to mesenteric lymphadenopathy ((**a**), red arrows) and multiple liver metastases (pink arrows), the most prominent one situated in the VIII hepatic segment (**b**), visible on the WB planar scan as well (**c**).

**Figure 3:**
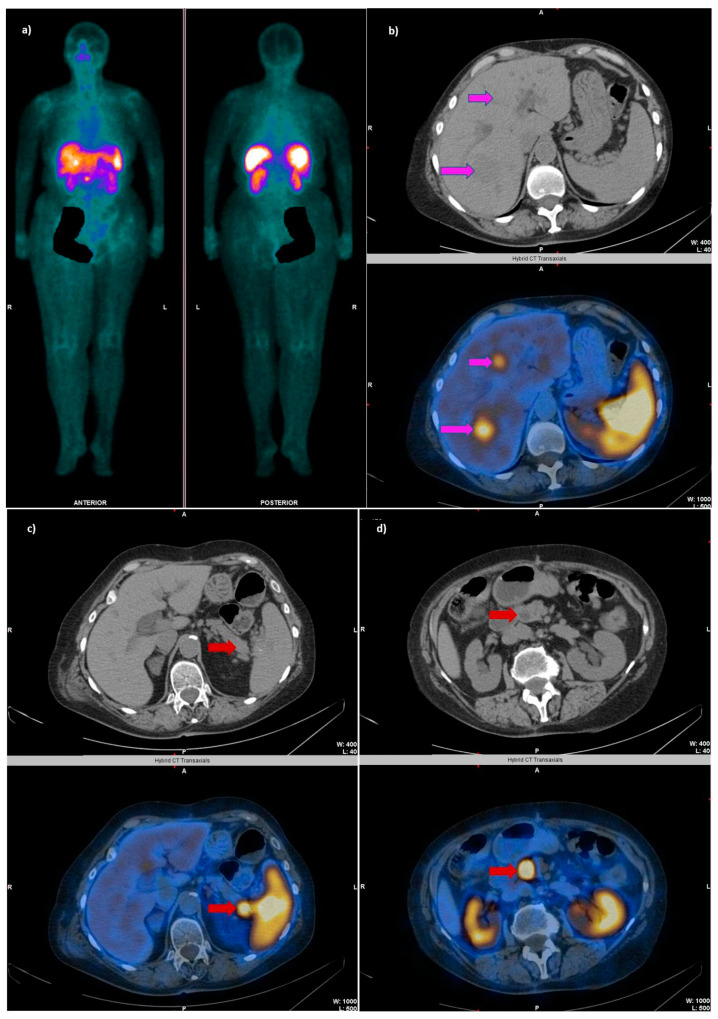
A 69-year-old female patient diagnosed with a pancreatic neuroendocrine tumor (confirmed through multiple biopsies from the pancreatic head and liver metastasis as NET G2), undergoing treatment with somatostatin analogs. The WB SRS revealed ^99m^Tc-EDDA/HYNIC-TOC uptake in the abdominal region (**a**), with the SPECT/CT showing SSTR-expressing liver metastases (**b**) and two foci of increased uptake in the pancreas: one corresponding to the tail of the pancreas (**c**) and one in the head of the pancreas (**d**).

Patients’ characteristics are resumed in Table 1. The most frequent tumoral site found in our patients was the pancreas (41%), followed closely by the small intestine (39.9%). Also, 98 patients (57%) had metastatic disease, the most common localizations being: the liver (46.2%), lymph nodes (32.9%), bones (5.8%), the peritoneum (4.1%), and lungs (2.9%) (Figure 2 and Figure 3). A number of 49 patients (28.5%) were undergoing treatment with long-acting somatostatin analogs at the moment of the investigation and were asked to interrupt it 4 weeks prior to SRS. The positivity rates for the neuroendocrine tumoral markers are presented in Table 2 and Table 3.

**Figure 4:**
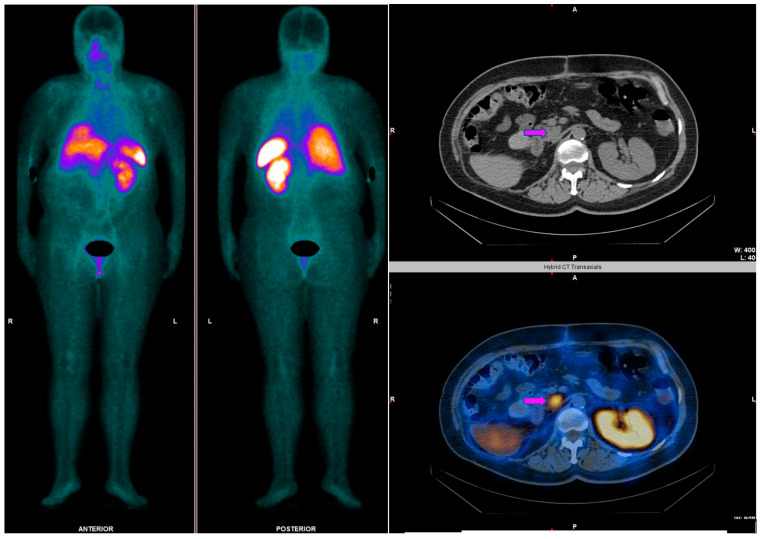
A 69-year-old female patient who underwent ^99m^Tc-EDDA/HYNIC-TOC SRS for biochemical suspicion of NET (high values of seric Chromogranin A). The scan revealed increased uptake in the uncinate process of the pancreas (arrow). The histopathological examination from the subsequent excision of the mass proved to be negative for NET.

Diagnosis was made according to both whole-body planar and SPECT/CT examinations and in concordance with results from previous CT, MRI, and endoscopic tests. Out of the 173 examinations performed, 121 were assessed as positive. True positive (TP) results were found in 105 cases, false positive (FP) results in 16 cases, true negative (TN) in 41 cases, and false negative (FN) in 11 cases. We considered as false positive findings uptakes in the uncinate process of the pancreas that proved to be negative for NET on further histopathological exams (Figure 4), in adrenal adenomas and in different inflammation sites (when they were previously diagnosed or the patient was known to have had recent surgery). Furthermore, when assessing the uptake in the uncinate process of the pancreas, we gauged as TP uptakes that presented a higher intensity than the liver and as FP the uptakes with a lower intensity than the one of the liver. We appraised as false negative findings the lack of ^99m^Tc-EDDA/HYNIC-TOC uptakes in patients with metastases diagnosed on other imaging methods, lack of fixation in restant tumoral tissue after tumor excision (especially in polypoid gastric lesions), and non-fixating insulinomas diagnosed otherwise. We estimated a sensitivity of the method of 90.5% and a specificity of 71.9%. The accuracy of ^99m^Tc-EDDA/HYNIC-TOC SRS was 84.3%, with a PPV of 86.7% and a NPV of 78.8% (Table 4).

We separately calculated the sensitivity and specificity for pancreatic NETs and obtained a value of 94.6% for sensitivity, respectively, of 73.3% for specificity, with an accuracy of 90.1%. The values obtained for gastrointestinal NETs were as follows: sensitivity of 86.7%, specificity of 71.4%, and accuracy of 80.3%.

## 4. Discussion

Somatostatin receptor scintigraphy, as a singular planar method or combined with SPECT/CT, has been long and widely used in clinical practice for diagnosis and monitoring of GEP-NETs. Although recently introduced on the market and not sufficiently researched yet, ^99m^Tc-EDDA/HYNIC-TOC could represent a good alternative to other, more commonly used radiotracers, such as ^111^In-pentetreotide and ^68^Ga-peptides, ^68^Ga-DOTATOC, ^68^Ga-DOTANOC, and ^68^Ga-DOTATATE [13]. The usage of SPECT/CT systems significantly improves the accuracy of the method and allows a better spatial resolution than SPECT-alone studies (7–9 mm) [22]. Moreover, even if ^68^Ga-DOTA-PET/CT is considered the “golden standard” in the diagnosis, treatment monitoring and management of patients suffering from neuroendocrine tumors and SPECT/CT using ^99m^Tc-EDDA/HYNIC-TOC might represent a great alternative in centers that do not benefit from PET equipment and in countries where ^68^Ge/^68^Ga generators are not available, as ^99m^Tc can be easily maneuvered, and its radiation burden in patients is relatively small. Our results demonstrate a very good diagnostic utility of ^99m^Tc-EDDA/HYNIC-TOC scintigraphy, with high sensitivity, specificity, accuracy, positive and negative predictive values, in concordance with outcomes obtained by other authors. Our sensitivity of 90.5% is higher than the ones obtained by other authors [23,24,25,26], but lower to the ones obtained in ^68^Ga-studies.

Several authors analyzed the efficacy of this technique as well. Saponjski et al. [23] studied the diagnostic and prognostic value of ^99m^Tc-EDDA/HYNIC-TOC SRS in comparison to ^18^F-FDG PET/CT in all types of NETs and concluded that ^18^F-FDG PET/CT had a higher sensitivity than SRS (92% vs. 83.6%), probably correlated to the higher number of patients with high tumoral-grade NETs (27 NET G2 tumors and 15 NET G3 tumors). The other values obtained for ^99m^Tc-EDDA/HYNIC-TOC SRS were a specificity of 82.6% and an accuracy of 83.3%. Artiko et al. [24], who included a large cohort of 495 patients, with predominantly GEP-NETs and NETs of unknown origin in their study, obtained an overall sensitivity of the method of 80%, with a specificity of 92%, PPV of 98%, NPV of 47%, and accuracy of 82%. Vlajković et al. [25] examined 61 patients with carcinoid tumors and discovered that the likelihood of a positive ^99m^Tc-EDDA/HYNIC-TOC SRS is inversely proportional to the value of ki67 proliferation index. They attained a sensitivity of 78.79%, specificity of 84.62%, PPV of 86.67% and NPV of 75.86%. Another group of authors, Kunikowska et al. [26], investigated 68 patients suffering predominantly of GEP-NETs, both with ^99m^Tc-EDDA/HYNIC-TOC SRS and ^68^Ga-DOTATATE PET/CT. They concluded that ^68^Ga-DOTATATE PET/CT has higher sensitivity (100% vs. 82%), specificity (85% vs. 69%), PPV (97% vs. 92%), NPV (100% vs. 47%), and accuracy (97% vs. 79%) than ^99m^Tc-EDDA/HYNIC-TOC scintigraphy, and also demonstrated that ^9m^Tc-EDDA/HYNIC-TOC and ^68^Ga-DOTATATE have different SSTR affinities. The difference between the values estimated by these groups of authors and our results might stem from the smaller variety of NET types analyzed in our study (GEP-NETs) that presented a high tumoral grade on histopathological examinations (NET G1-G2), which are more abundant in SSTRs, and from the examination protocol used in our study: a whole-body planar acquisition made 2 h after ^99m^Tc-EDDA/HYNIC-TOC administration and a SPECT/CT scan made 4 h after the injected dose, resulting in a different observable biodistribution of the radiotracer than in scans performed even after 24 h.

**Figure 5:**
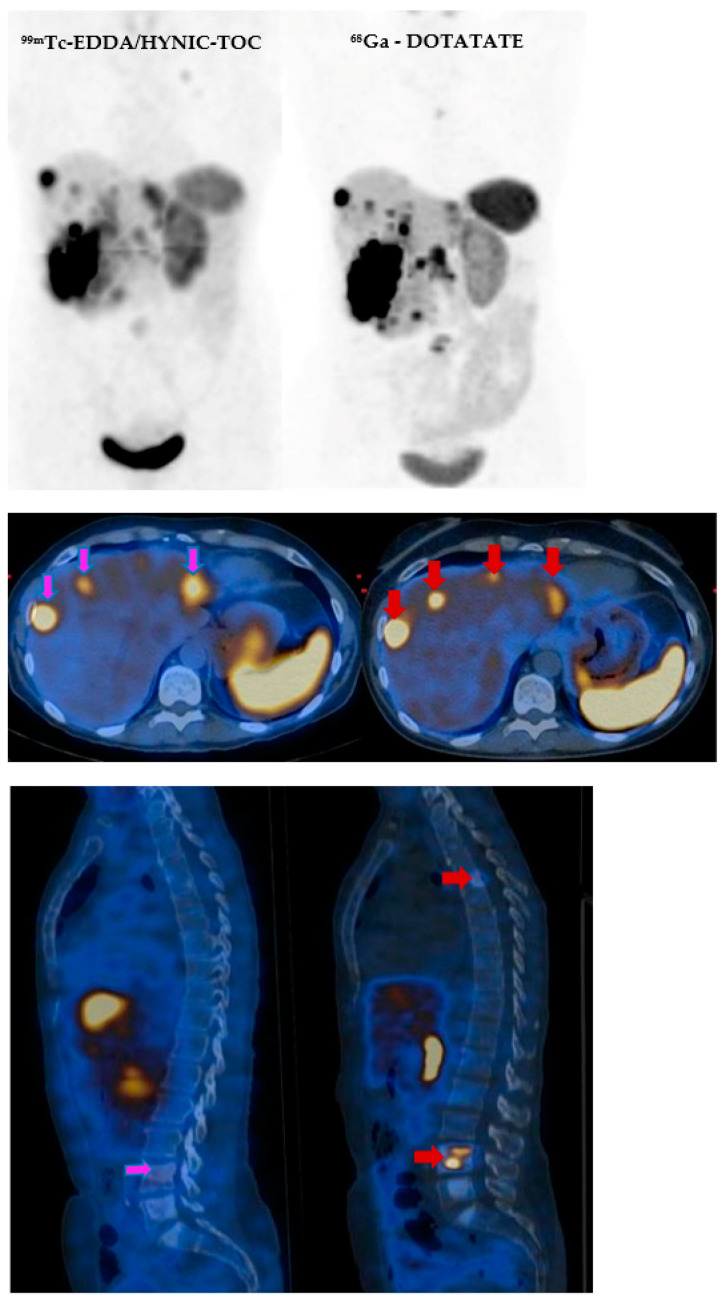
Comparison between ^99m^Tc-EDDA/HYNIC-TOC SPECT/CT (pink arrows) and ^68^Ga-DOTATATE PET/CT (red arrows) in a 54-year-old female patient, diagnosed with a pancreatic neuroendocrine tumor (G2), with multiple liver, lymphatic, and osseous metastases. The patient underwent four sessions of ^177^Lu-DOTATATE PRRT between the PET scan and the SPECT examination. A better resolution for the ^68^Ga-DOTATATE study can be observed, highlighting its higher capacity to detect smaller hepatic lesions and bone metastases. However, similar results can be seen both on ^99m^Tc-EDDA/HYNIC-TOC SPECT/CT and ^68^Ga-DOTATATE PET/CT for bigger lesions.

A meta-analysis published by Deppen et al. [27] estimated a pooled sensitivity and specificity of ^68^Ga-DOTATATE of 90.9% (95% CI, 81.4–96.4%), respectively, of 90.6% (95% CI, 77.8–96.1%) in patients with GEP-NETs and lung NETs gathered from 10 papers. Another review made by Lee et al. [16] estimated a mean sensitivity of 92% (range 68–100%) and a mean specificity of 88% (range 50–100%) of ^68^Ga-DOTA-peptides PET/CT in patients with pancreatic NETs. These pooled results, combined with the higher spatial resolution of PET/CT systems (4–5 mm vs. 7–9 mm in SPECT/CT), confirm the superiority of ^68^Ga-peptides in diagnosis of GEP-NETs, presenting a greater sensitivity and specificity than the ones obtained using ^99m^Tc-EDDA/HYNIC-TOC; however, both ^68^Ga-DOTATATE and ^99m^Tc-EDDA/HYNIC-TOC can present a significant utility for conventional treatment monitoring or PRRT planning, with good results (Figure 5) [22,26].

When compared to ^111^In-pentetreotide studies, ^99m^Tc-EDDA/HYNIC-TOC has ideal imaging characteristics, showing better results with SPECT/CT systems for correct characterization and localization of metastases and primary lesions [28]. It has also been stated that more liver metastases are detected using ^99m^Tc-labeled somatostatin analogs as compared to ^111^In-pentetreotide [29]. Lee et al. [16] state in their review that the overall sensitivity of ^111^In-pentetreotide was 60–80%, with an overall specificity that ranged from 92–100%. Even when using SPECT/CT, ^111^In-pentetreotide SRS demonstrates a lower sensitivity (54%) compared to the other available radiotracer options [30]. This makes ^99m^Tc-EDDA/HYNIC-TOC a more significant and available alternative to the usual ^111^In studies.

There are, however, several limitations to our study. First of all, histopathological confirmation was not available for all the lesions observed, allowing the possibility of false positive results to be included in the analysis, for example uptake in inflammatory lymph nodes. Secondly, the SPECT/CT resolution is still lower than that of a conventional PET/CT system (7–9 mm vs. 4–5 mm), giving the possibility of false negative results through the difficulty of assessing small lesions.

## 5. Conclusions

^99m^Tc-EDDA/HYNIC-TOC, a receptor-based radiopharmaceutical could represent a good alternative to ^68^Ga-labeled peptides in the diagnosis and management of patients with gastroenteropancreatic neuroendocrine tumors. Our results show a lower sensitivity (90.5%) than ^68^Ga-DOTA-peptides, but with good specificity, accuracy, and positive and negative predictive values.

Given the continuous development of the SPECT/CT and the software used to analyze the obtained data, ^99m^Tc-EDDA/HYNIC-TOC SRS offers prospects for treatment monitoring through SUV-based evaluations, that could represent a cheaper and widely available method for assessing disease progression and recovery.

## Figures and Tables

**Table 1:** Patients’ characteristics.

Age (Mean ± SD)	55 ± 13.36		
		Number	Percentage
**Sex**	F	98	56.6%
	M	75	43.4%
**NET localization**	Stomach	20	11.6%
	Small Bowel	69	39.9%
	Large Bowel	9	5.2%
	Appendix	4	2.3%
	Pancreas	71	41%
**Tumor grade**	NET G1	81	46.8%
	NET G2	62	35.8%
	NET G3	3	1.7%
	NA	27	15.6%

Abbreviations: F—female; M—male; NET—neuroendocrine tumors; NA—not applicable.

**Table 2:** Positivity rate for neuroendocrine immunohistochemical markers.

Immunohistochemical Marker	Positivity Rate (%)
CGA	78.6
SYN	66.5
CDX2	12.7
NSE	16.8
CD56	16.2

Abbreviations: CGA—Chromogranin A; SYN—Synaptophysin; CDX2—Caudal type homeobox transcription factor 2; NSE—Neuron Specific Enolase; CD56—homophilic binding protein.

**Table 3:** Positivity rate for neuroendocrine biochemical markers.

Biochemical Marker	Positivity Rate (%)
Serotonin	23.1
Gastrin	9.9
Insulin	1.7
Glucagon	1.7
5-HIAA	11

Abbreviations: 5-HIAA—5 Hydroxyindolacetic acid.

**Table 4:** Characteristics of ^99m^Tc-EDDA/HYNIC-TOC SRS in our population.

	Obtained Value
Sensitivity	90.5%
Specificity	71.9%
Accuracy	84.3%
PPV	86.7%
NPV	78.8%

Abbreviations: PPV—positive predictive value; NPV—negative predictive value.

## Data Availability

All the data generated or analyzed during this study are included in the manuscript.
